# Characterization of the invariable residue 51 mutations of human immunodeficiency virus type 1 capsid protein on in vitro CA assembly and infectivity

**DOI:** 10.1186/1742-4690-4-69

**Published:** 2007-09-28

**Authors:** Samir Abdurahman, Masoud Youssefi, Stefan Höglund, Anders Vahlne

**Affiliations:** 1 Division of Clinical Virology, Karolinska Institutet, F68 Karolinska University Hospital, SE-141 86 Stockholm, Sweden; 2 Department of Biochemistry, Uppsala University, Uppsala, Sweden

## Abstract

**Background:**

The mature HIV-1 conical core formation proceeds through highly regulated protease cleavage of the Gag precursor, which ultimately leads to substantial rearrangements of the capsid (CAp24) molecule involving both inter- and intra-molecular contacts of the CAp24 molecules. In this aspect, Asp51 which is located in the N-terminal domain of HIV-1 CAp24 plays an important role by forming a salt-bridge with the free imino terminus Pro1 following proteolytic cleavage and liberation of the CAp24 protein from the Pr55Gag precursor. Thus, previous substitution mutation of Asp51 to alanine (D51A) has shown to be lethal and that this invariable residue was found essential for tube formation in vitro, virus replication and virus capsid formation.

**Results:**

We extended the above investigation by introducing three different D51 substitution mutations (D51N, D51E, and D51Q) into both prokaryotic and eukaryotic expression systems and studied their effects on in vitro capsid assembly and virus infectivity. Two substitution mutations (D51E and D51N) had no substantial effect on in vitro capsid assembly, yet they impaired viral infectivity and particle production. In contrast, the D51Q mutant was defective both for in vitro capsid assembly and for virus replication in cell culture.

**Conclusion:**

These results show that substitutions of D51 with glutamate, glutamine, or asparagine, three amino acid residues that are structurally related to aspartate, could partially rescue both in vitro capsid assembly and intra-cellular CAp24 production but not replication of the virus in cultured cells.

## Background

The HIV-1 Pr55Gag precursor, which comprises the inner structural proteins of the virus, is sufficient for assembly of retrovirus-like particles in mammalian cells. During HIV-1 assembly and maturation, the transformation of the virus from a spherical to a conical core structure results as a consequence of substantial inter- and intra-molecular rearrangements of one of the Pr55Gag derived proteins, namely the capsid protein (CAp24). This process is initially driven by the viral protease which sequentially cleaves Pr55Gag and liberates the mature structural proteins that forms the viral core structure [1,2]. The mature conical HIV-1 core, which is composed of approximately 1500 CAp24 molecules [3], is comprised of two independently folded subunits, the N- and C-terminal domains (NTD and CTD) [4]. The N-terminal domains of CAp24 are assembled into hexameric rings [5] and each hexameric ring is joined to the neighbouring ring by the CTDs of CAp24 resulting in a lattice with local p6 symmetry.

**Figure 1:**
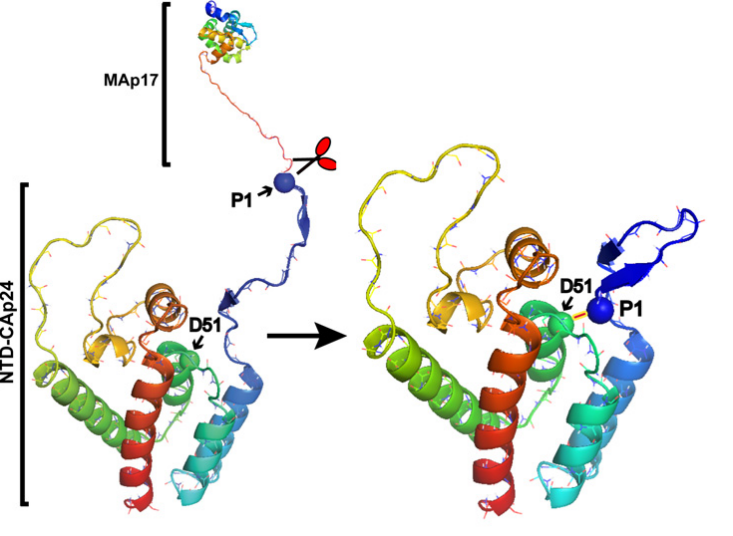
**Ribbon representation showing the MAp17 and the N-terminal CAp24 domain of the unprocessed Pr55Gag**. Ribbon diagram of the MAp17 [33] and CAp24 [34] depicting the structural rearrangemts that takes place in the N-terminal domain (NTD) of CAp24 upon proteolytic processing at the MAp17-CAp24 junction (indicated with a sax). The model to the right represents a processed NTD CAp24 showing the β-hairpin formation which is stabilized by the salt-bridge formation between the imino terminal Pro 1 and Asp 51. For clarity, Por 1 and Asp 51 are shown as filled circles. The ribbon diagrams were generated with the PyMOL [35] and modified with Adobe Photoshop software.

The availability of high resolution structures combined with mutagenesis studies of the HIV-1 CAp24 have provided important insights on the structure and mechanisms of virus assembly. Using these biological techniques, the importance of Asp51 in the NTD of CAp24 has been described before [6]. The study showed that mutation of Asp51 to alanine to be lethal. Thus, this invariable residue was shown to be essential for CAp24 tube formation in vitro, and for HIV-1 replication and capsid formation in cultured virus [6]. During proteolysis of the Pr55Gag and maturation of CAp24, the NTD of CAp24 refolds into a β-hairpin structure which is then stabilized by formation of a salt-bridge between Pro1 and Asp51 of the processed NTD (Fig. 1). The fact that this structure is not formed in immature virus-like structures [7] also indicates that this motif does not form in an immature particle. The importance of this structure is further emphasized by the fact that all mature retroviral capsids, with possible exception of foamy virus, contain an N-terminal β-hairpin loop. In the case of murine leukemia virus for example, a virus which belongs to a gamma-retrovirus family, Pro1 forms a salt-bridge with a highly conserved Asp54, which is the equivalent to Asp51 in HIV [8]. A high degree of conservation among residues involved in formation and stabilization of this structure also exists in various retroviruses. In multiple sequence alignment analysis of 4198 HIV-1 CAp24 sequences found in the HIV database (May 7, 2007), we found only 11 exceptions to the highly conserved Asp51 among all HIV-1 strains, demonstrating that this residue is not only conserved among various retroviruses but also in HIV strains.

Since mutation of Asp51 to alanine has shown to be critical for proper capsid formation and subsequent replication of the virus, we extended the above findings and examined amino acid substitutions of this invariable residue to asparagine, glutamate, and glutamine. All three amino acid residues closely resemble aspartate and were anticipated not to grossly interrupt the CAp24 structure. We designed the mutated Cap24 sequences in both prokaryotic and eukaryotic expression systems and studied their effects in vitro, as well as, in vivo. Two of the three mutants (D51E and D51N) were stable in vitro as was evidenced by forming highly polymerized capsid tubular structures that were closely resembling wild type structure, however, the infectivity and in vivo morphological structures of all three mutants were severely affected.

## Results

### Viral protein expression of HIV-1 CAp24 mutants

**Figure 2:**
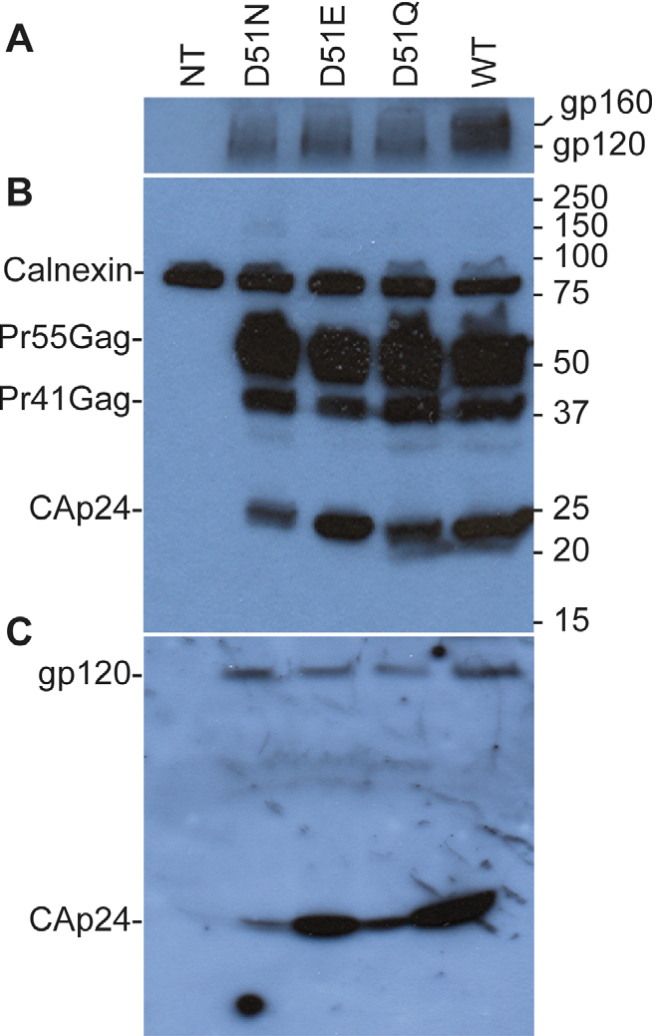
**Western blot analysis of transfected HeLa-tat cell and precipitated viruses**. HeLa-tat cells were transfected with the plasmids indicated using the non-liposomal transfection reagent. Forty-eight hrs post-transfection, cells were washed and harvested in 1× RIPA buffer. Particles released into the culture supernatant were also clarified and filtered of cell debris and precipitated with Viraffinity (CPG) as recommended by the manufacturer. Denatured cell (A and B) and viral lysates (C) were then separated by SDS-PAGE, transferred onto a nitrocellulose membrane and detected with a rabbit anti-HIV glycoprotein (A), a pool of anti-CAp24 and anti-calnexin (B), and anti-CAp24 (C) antibodies. The positions of specific viral proteins are indicated to the left and the numbers to the right depict positions of molecular mass markers (in kDa). NT, a mock control; WT, wild type; and D51N, D51E, and D51Q are the three CAp24 mutants.

We investigated the effects of three HIV-1 CAp24 mutants carrying the D51N, D51E, and D51Q mutations for viral protein expression by initially transfecting HeLa-tat cells. Total cell lysates were immunoblotted and detected with polyclonal antibodies directed against gp120/gp160 (Figure 2A), a pool of antibodies against CAp24 and calnexin (Figure 2B), and precipitated viral lysates were immunoblotted with a pool of HIV-positive sera from two individuals (Figure 2C). Two to three days post-transfection, processed HIV-1 Pr55Gag proteins were detected in all cell lysates. The relative intracellular level of the Pr55Gag precursor in all mutants was comparable to that of the wild type, whilst the D51N and D51Q mutants displayed somewhat reduced levels of the CAp24. Whereas the D51Q mutant displayed a slightly reduced amount of CAp24, the level of processed CAp24 proteins in the D51N mutant was significantly reduced relative to the wild type and the D51E CAp24 mutant. To further evaluate the level of viral proteins in released virions, normalized amounts of culture supernatants were precipitated with Viraffinity and detected with immunoblotting using both monoclonal and polyclonal anti-CAp24 antibodies (data not shown) and a pool of HIV-positive sera from two individuals (Figure 2C). Mature CAp24 represented the major product of the precipitated material. However, the level of this protein in both D51N and D51Q mutants was significantly reduced relative to the wild type and D51E mutant, correlating with the lower intracellular CAp24 levels. A comparable level of the viral glycoprotein (gp120) incorporation into released virions was observed with all mutants and the wild type virus (Figure 2C). A similar result was also obtained with a V3 loop-specific monoclonal anti-glycoprotein antibody (data not shown).

**Figure 3:**
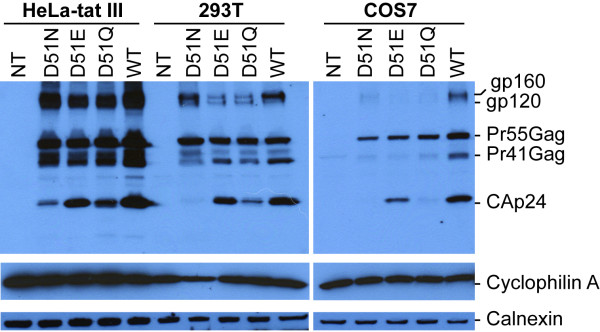
**Western blot analysis of cell-type dependent expression of HIV-1 proteins**. HeLa-tat III, 293T and COS7 cells were transfected as described above with mutant and wild type proviral DNA constructs. Forty-eight hrs post-transfection, cells were washed and harvested in 1× RIPA buffer. Denatured cell lysates were then resolved by SDS-PAGE, transferred to a nitrocellulose membrane and immunoblotted with a pool of two HIV-1 positive sera (A), rabbit anti-cyclophilin A (B), and anti-calnexin (C) antibodies. Positions of specific viral and cellular proteins are indicated on the right.

The Pr55Gag expression and processing pattern was further characterized by transfecting HeLa-tat III, 293T and COS7 cells with the wild type and mutant pNL4-3 expression plasmids and detected with immunoblotting using a pool of HIV-positive human sera from two individuals (Figure 3). With HeLa-tat III cells (Figure 3), the levels of CAp24 detected with the D51N and D51Q were largely identical with those in HeLa-tat cells detected with a rabbit anti-CAp24 antibody (Figure 2B). Additionally, fully processed Pr55Gag proteins, as well as, the surface glycoproteins could be detected with all mutants when using a pool of HIV-positive human sera. Further reduction or absence of cell-associated CAp24 of the D51N and D51Q mutants was observed in both 293T and COS7 cells. Whereas no CAp24 was detected with the D51N mutant, significantly reduced level of this protein was observed with the D51Q mutant in both 293T and COS7 cells. Similar results were also obtained when using both monoclonal and polyclonal antibodies directed against CAp24 or the surface glycoprotein gp120/gp160, respectively (data not shown). With the wild type control, fully processed HIV-1 Gag proteins were detected in all three transfected cell lines. As an internal control, the level of cell associated cyclophilin A and calnexin were probed with polyclonal antibodies directed against these two proteins (Figure 3, lower panels).

### In vitro CAp24 assembly

**Figure 4:**
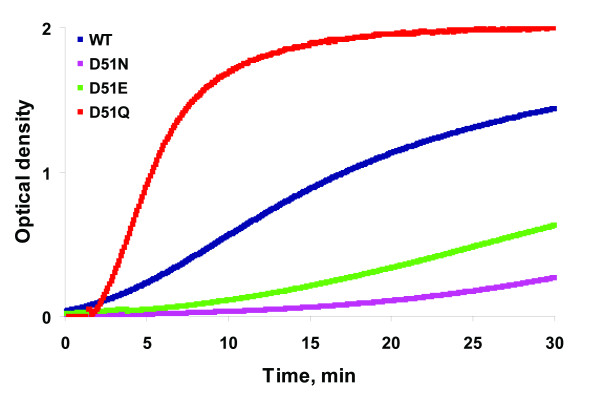
**Turbidity assay showing the effects of CAp24 mutations on in vitro CA assembly**. Turbidity assay showing the increase in light absorbance after addition of 2.0 M NaCl to recombinantly produced mutant and wild type CAp24 protein (100 μM) reflecting the assembly of the CAp24 protein into tubular structures. Green, D51E; red, D51Q; blue, wild type; pink, D51N. The structures of polymerized CAp24 structures were also analyzed by transmission electron microscopy (Figure 5).

Turbidity assay is a valuable technique used to study a salt-induced self-assembly process of CAp24 by monitoring polymerization of CAp24 spectrophotometrically, as the rate of CAp24 tube formation can be seen as an increase in sample turbidity over time. One-hundred μM of each CAp24 was mixed with NaH_2_PO_4 _(pH 8.0) buffer and polymerization was induced by addition of concentrated NaCl solution. The rate of CAp24 tube formation was then measured spectrophotometrically (at 350 nm) over time. As shown in Figure 4, an increase in sample turbidity was observed for both D51N and D51E mutant CAp24 proteins. However, as expected, the kinetics of CAp24 assembly was lower than that of the wild type control. In marked contrast, the rate of sample turbidity increase for the D51Q mutant CAp24 was higher than for the wild type control. This was quite surprising to us, as the increase in OD should be proportional to the total number of CAp24 proteins assembled into tubular structures [9].

### Morphological analysis of structures formed by recombinant HIV CAp24 in vitro

**Figure 5:**
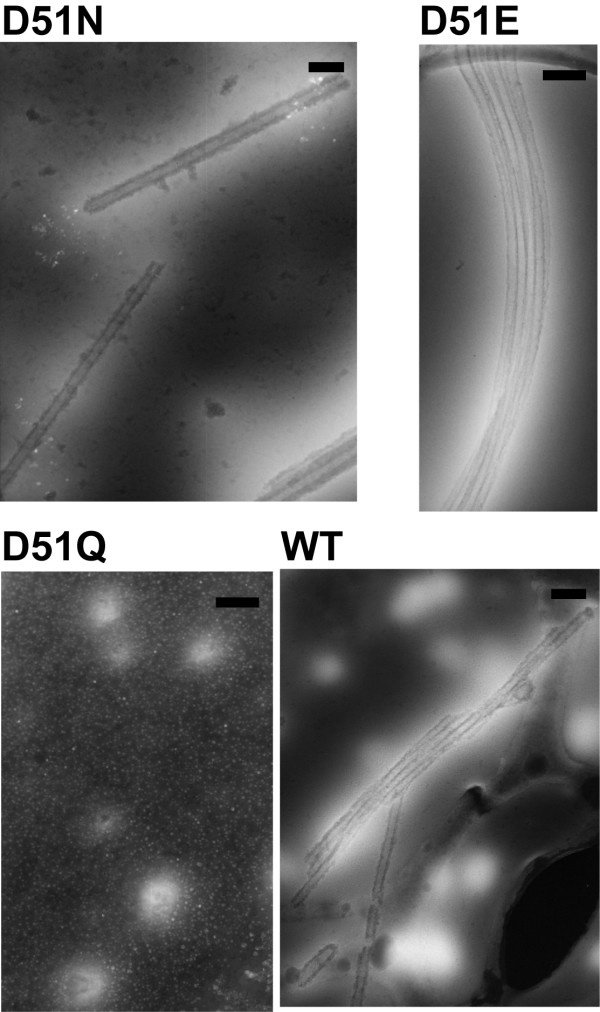
**Morphological analysis of in vitro assembled mutant CAp24 proteins**. Mutant and wild type CAp24 proteins were induced for in vitro CAp24 tubular formation (see Fig. 3). At the end of the experiment, the proteins were fixed in freshly prepared 2.5% glutaraldehyde. The electron micrographs show negatively stained thin-sections of the in vitro assembled CAp24 tubular structures used in turbidity assay. Micrographs of the CAp24 mutant D51N (A), D51E (B), D51Q (C) and the wild type CAp24 (D). Bars indicate 100 nm.

To determine the effects of CAp24 mutations on in vitro capsid assembly, thin-sections of the polymerized material used in turbidity assay was prepared and analyzed by transmission electron microscopy. As shown in Figure 5, long tubular structures were observed in both D51N and D51E mutant CAp24 proteins induced by addition of 2.0 M NaCl solution. Additionally, the morphology of the tubes formed by these two was comparable to the structures formed by wild type CAp24, both in terms of external diameter and length of the tubes. In contrast, no structure that resembled CAp24 tubular formation was observed with the D51Q mutant CAp24 protein under the same conditions.

### Analysis of virus release and infectivity

**Figure 6:**
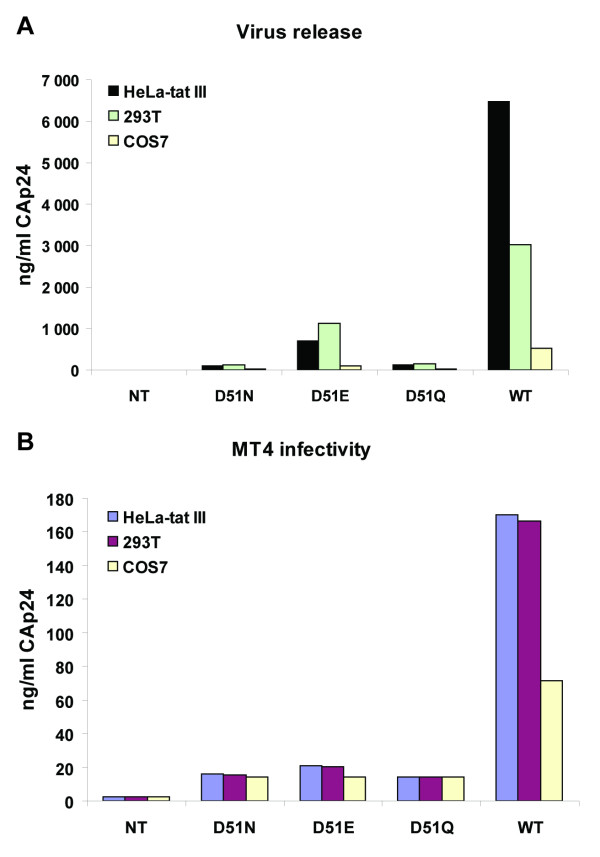
**Virus release from transfected cells and their infectivity**. HeLa-tat III, 293T, and COS7 cells were transfected with mutant and wild type proviral DNAs as indicated. (A) Three days post-transfection, culture supernatants were collected and analyzed by CAp24-ELISA. (B) Normalized amounts of cleared and filtered culture supernatants from the above transfected cells were then used to infect MT4 cells (1 × 10^5 ^cells per well in 48-well plate) using 100 ng of CAp24 antigen. The bars indicate infectivity of the virus particles produced from the three different cell lines monitored by CAp24-ELISA.

The effects of CAp24 mutations on Pr55Gag assembly and virus particle release was also analyzed by measuring the CAp24 antigen contents released into the culture medium of transfected HeLa-tat III, 293T and COS7 cells. As shown in Figure 6A, the CAp24 antigen levels in the culture supernatant of D51N and D51Q transfected cells were negligible in all three cell lines, whereas the virus production of the D51E mutant was reduced by 2- to 6-fold as compared to the wild type.

The effect of the three CAp24 mutations on virus infectivity was then assessed with culture supernatants from transfected HeLa-tat III, 293T and COS7 cells. MT4 cells were infected with equal amount of cleared and filtered culture supernatants (normalized for CAp24 antigen) and assayed for CAp24 antigen contents with a CAp24-ELISA three days post-infection (Figure 6B). While none of the three mutant viruses were able to replicate, as expected, the wild type virus replicated in this cell line. Similar results were also seen when the infectivity of mutant viruses was tested in H9 cells (data not shown). We kept the infected H9 cell cultures for more than 25 days without detecting virus replication with the mutants. No revertants to wild type virus were observed.

### Single cell cycle infectivity of HIV-1 CAp24 mutant virions

**Figure 7:**
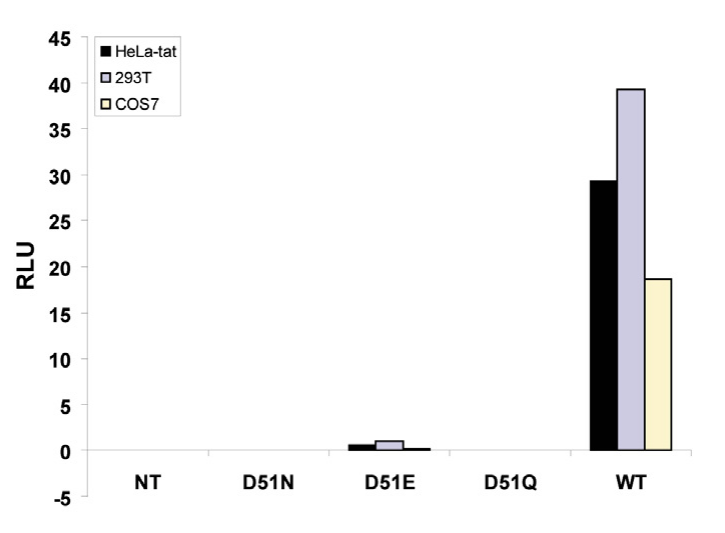
**Single cell cycle infectivity of mutant and wild type virus particles on TZM-bl reporter cell lines**. For relative viral infectivity assay, TZM-bl reporter cell lines were seeded one day before infection. Following day, medium was removed and target cells were inoculated by adding equal amounts of mutant and wild type NL4-3 virus produced from transfected HeLa-tat III, 293T, and COS7 cells. In this assay, expression of the reporter luciferase gene is under the control of Tat protein that is activated by Tat protein synthesized from the infecting virus. Infected cells were then analyzed 24 hrs post-infection with the luciferase assay kit obtained from Promega and as recommended by the manufacturer. RLU, relative light unit.

Since the infectivity of all three CAp24 mutants were reduced or completely absent when assayed in MT4 cells, we analyzed the infectivity of these viruses produced from three different cell lines in a single cell cycle infectivity assay using the TZM-bl reporter cell line [10]. In this assay, expression of the reporter luciferase gene is under the control of Tat protein that is activated by Tat protein synthesized from the infecting virus. While the Tat-induced luciferase activity could not be detected in cells infected with mutant D51N and D51Q virions, only a subtle amount of luciferase activity was observed repeatedly in cells infected with the D51E virions (Figure 7). On the other hand, the level of Tat-induced luciferase activity was significantly higher in cells infected with the wild type virus.

### Immunofluorescence analysis of viral protein expression in transfected cells

**Figure 8:**
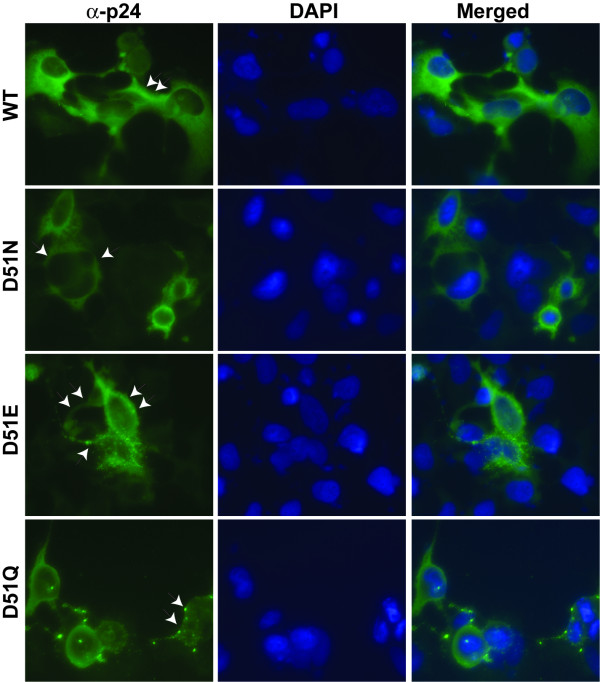
**Immunofluorescence analysis of transfected HeLa-tat III cells**. HeLa-tat III cells were transfected with mutant and wild type proviral DNA constructs. Forty-eight hrs post-transfection, cells were fixed and stained with a mouse anti-CAp24 monoclonal antibody. As a secondary antibody, FITC-conjugated (green) rabbit anti-mouse IgG was used. DAPI (4',6-diamidino-2-phenylindole dihydrochloride) was used to stain cell nuclei. The images in the right column represent an overlay of anti-CAp24 and DAPI stained images.

The viral protein expression profiles were further investigated by their staining patterns using monoclonal antibody directed against CAp24. All mutants displayed strong specific signals (indicated with arrows in Figure 8) concentrated near or at the plasma membrane. This feature was most pronounced in cells transfected with the three capsid mutants and not with the wild type pNL4-3 transfected cells. The staining pattern seen with the wild type control was mostly throughout the whole cytoplasm and the plasma membrane (Figure 8, panel WT). A representative staining pattern of each mutant and the wild type control is shown.

### Effect of HIV-1 CAp24 mutations on virion morphology

**Figure 9:**
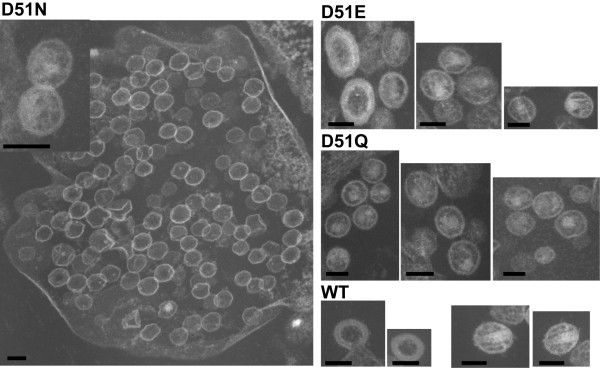
**TEM analysis of mutant virus particles**. Electron micrographs of mutant and wild type virus particles. Mutants D51N and D51Q showed mostly heterogenous populations of particles with varying size and morphology (panels D51Q and inset in panel D51N). No virus particles with conical core structures were observed with these two mutants. Additionally, a large pool of virus-like structures inside vesicles released from transfected cells were observed in D51N mutant. With the wild type and D51E virions particles representing immature-like viruses are shown (panels D51E and WT). Mature viruses with conical structures were seen only in the wild type control virus. Occasionally, D51E virions resembling the mature wild type morphology but with aberrant core structure was also observed. Bars indicate 100 nm.

Morphogenesis of all mutant viruses and the wild type control were analyzed by transmission electron microscopy. The D51N and D51Q mutant virions showed mostly particles devoid of the typical HIV-1 core structure (Figure 9, panel D51N and D51Q). Instead, heterogeneous virus populations with aberrant core structures were observed. Additionally, the D51N virions showed a large pool of intra-vesicular viruses that were deficient of the electron dense core structure. Most strikingly, no mature virus particles with conical core structures were detected with these two mutants. A limited number of immature-like viruses and occasionally mature-like viruses but with aberrant cores were observed with the D51E mutant. Only the wild type control produced viruses with typical immature- and mature-like HIV-1 virions (Figure 9, panel WT). Similar results were also observed when virus infected Jurkat-tat cells were analyzed (data not shown).

## Discussion

Proper structural rearrangement of capsid (CAp24) after Pr55Gag cleavage is a highly conserved feature in most retroviruses [11]. As a result of this process, a β-hairpin structure formed by a salt-bridge between Pro1 and Asp51 (D51) of HIV-1 is important for conformational stability of the N-terminal CAp24 structure [6]. Thus, mutations of D51 in HIV-1 CAp24, and likewise Asp54 in murine leukemia virus (MLV) or human T-cell leukemia virus-1 (HTLV-1), has been shown to disrupt formation of this β-hairpin structure [6,8,12].

Structural and mutagenesis studies of D51A mutation in HIV-1 CAp24 has previously shown this invariable residue to be essential for tube formation in vitro, and for the replication and capsid formation in cultured virus [6]. We here demonstrated that substitution of D51 with glutamate (D51E), asparagine (D51N), but not glutamine (D51Q) (three amino acids which in proteins have similar properties as aspartate; Glu > Asn > Gln) could partly restore in vitro CAp24 assembly but not the infectivity of the virus particles.

Whereas generally the total protein contents produced by transfected 293T and COS7 cells were reduced as compared to HeLa-tat or HeLa-tat III cells, similar Pr55Gag-processing patterns was repeatedly observed in all mutant and wild type proviral DNA transfected cells. However, intracellular concentrations of CAp24 protein in any of the cells transfected with D51N and D51Q were generally reduced. This could not be explained by the lack of recognition by the antibody used for immunoblotting, since detection with mouse anti-CAp24, rabbit anti-CAp24 or a pool of sera from HIV-infected patients gave similar results. Additionally, analysis with CAp24-ELISA using a different rabbit anti-CAp24-specific antibody also gave similar results. TEM analysis revealed that all mutants were assembly competent but produced virus particles with aberrant core morphology. The virus particles were also able to incorporate HIV-1 glycoprotein but the infectivity of the virus particles was severely reduced or absent suggesting that there was no defect at binding or internalization of these mutants although this was not specifically tested for. Whereas no infectivity was observed with the D51N and D51Q virions, a subtle amount was seen with the D51E viruses in a single cell cycle infectivity assay.

Further analysis of cytoplasmic versus cell membrane CAp24 distribution was also performed with indirect immunofluorescence staining using mouse anti-CAp24 antibody. This analysis revealed a strong staining pattern near or at the plasma membrane (PM) of cells transfected with the three mutants, indicating that there was no defect in intracellular transport of the Pr55Gag precursor to its steady-state destination [13] where activation of the viral protease takes place [14,15]. However, all mutants displayed a decreased cytoplasmic staining as compared to the wild type CAp24 control, which showed a diffuse cytoplasmic staining of non-membrane bound Pr55Gag/CAp24. Perhaps mutated Pr55Gag trafficking and/or assembly is slowed down, or even blocked close to or at the PM in agreement with low levels of mutant particles released. It is also possible that the virus release may have been blocked as a result of inability to form the stabilizing β-hairpin structure in the N-terminal domain of CAp24 upon proteolytic maturation which is necessary for assembly and release of virions [6].

Self-associative properties of many viral CAp24 proteins have been previously reported [16-19]. However, depending on the protein concentration, salt, and the buffering pH [9,20,21], the morphology of the assembled structures or the rate of assembly may be variable. The effects of D51 mutations on in vitro CAp24 assembly was monitored spectrophotometrically, and as expected, the assembly rate of both D51N and D51E mutants were substantially reduced relative to the wild type protein, although the ability of these mutants to form tubular structures was shown by thin-section transmission electron microscopy (TEM). Thus, it seems likely that the D51N and D51E mutations impose less structural changes than the D51A mutation described earlier [6]. Remarkably, although no tubular structure was observed with the D51Q mutant by TEM analysis, an increased optical density measurement that reflects the assembly kinetics was repeatedly observed. We cannot explain this, but, it is possible that the increased OD may result as a consequence of amorphous aggregates that are resistant for stable higher-order CAp24 tube formation.

In a recent study that was published after the present work was performed, Leschonsky et al [22] described the two single amino acid substitution mutations, a D183E and D183N, in an infectious provirus clone HX10. In contrast to our results, they found no effect on extracellular level of the CAp24 protein produced from H1299 cells transfected with the D183E mutant. Additionally, they found no effect on the intracellular level of the CAp24 protein in H1299 cells transfected with the D51N mutant. This may have been owing to the different cell type used. However, we analyzed the viral protein expression profiles in four different cell lines and found similar results.

Lastly, in order to correlate the lack of infectivity with morphological appearances of the viruses, electron microscopy analysis was performed. Only the D51E mutant particles were partially able to form immature- and mature-like viruses that resembled the wild type morphology. Importantly, despite the ability to form wild type-like viruses, the infectivity of D51E virions was significantly reduced, indicating the importance of optimal HIV-1 core stability [23]. With the two other non-infectious mutants, particles with aberrant core structures, either hollow-shaped spherical structures in endosomal vesicles (D51N) or particles with distorted core morphology (D51Q) were seen.

Taken together, our data and the other previously published observations [6,22,24] suggest that the invariable D51 residue of HIV is crucial for formation of the β-hairpin structure in matured CAp24 protein. Additionally, even substitution of D51 with such a similar residue as with glutamate could not restore the integrity of this structure. Furthermore, although our results demonstrated that the D51N and D51E substitutions could restore the in vitro tubular formation, the infectivity of all D51 mutation were rendered non-infectious indicating that this residue is indispensable.

## Methods

### Cells and reagents

HeLa-tat, 293T, COS7, and TZM-bl cell lines were maintained in Dulbecco modified Eagle medium (DMEM) supplemented with 10% fetal bovine serum (FBS), penicillin and streptomycin sulphate (Sigma, St Louis MO). H9, Jurkat-tat and MT4 cells were maintained in RPMI 1640 medium (Gibco, Grand Island, NY) supplemented with 10% fetal bovine serum (FBS; GIBCO), penicillin (100 U/ml), and streptomycin (100 μg/ml). DEAE-dextran was purchased from Sigma, rabbit polyclonal antibodies against calnexin from Santa Cruz Biotechnology (catalogue no. sc-11397). The following reagents were obtained through the AIDS Research and Reference Reagent Program, Division of AIDS, NIAID, NIH: All adherent cell lines, the protease inhibitor indinavir sulphate (catalogue no. 8145) and TZM-bl cells (catalogue no. 8129) contributed by Dr. John C Kappes [10].

### Plasmid DNA construction

The polymerase chain reaction (PCR) was utilized to develop all plasmids in the study and all constructs were derivatives of the HIV-1 molecular clone pNL4-3 [25]. The HIV-1 *CA *coding sequence was amplified using PCR and cloned into the prokaryotic expression vector pET11a (Novagen Inc.) essentially as described elsewhere [21,26]. Briefly, the primer pair 5'-AT**G GAT CCA TAT G**CC TAT AGT GCA GAA CCT CC-3' and 5'-AT**G GAT CC**T ATC ACA AAA CTC TTG CTT TAT GGC C-3' containing the *BamH*I/*Nde*I and *BamH*I, respectively, were used for amplification of the *CA *sequence (*Bam*HI/*Nde*I and *BamH*I sites are shown in bold). In addition, a translational start codon at the 5' end (ATG) and two stop codons (TGA/TAG) at the 3' end of the sequence were added. The PCR product was subcloned into the TA cloning vector (Invitrogen), transformed in DH5α *E. coli *(*Escherichia coli*), purified and confirmed by sequencing (Cybergene, Sweden). The vector was then digested with *Nde*I and *Bam*HI and the DNA fragment encoding *CA *gene was isolated, purified and cloned directionally into the pET11a vector, digested with the same restriction enzymes. Standard procedures were used for restriction digestion. The resulting plasmid was designated pET11a-CA and verified by sequencing.

The three HIV-1 CAp24 mutants, D51N, D51E, and D51Q, in the pET11a-CA vector were then engineered by site-directed mutagenesis using the Stratagene's QuickChange™ Site Directed Mutagenesis Kit (Stratagene) as recommended by the manufacturer. The primer pair used for creating the mutations is listed in Table 1.

**Table 1:** Primers used to create the D51N, D51E and D51Q CAp24 mutants

	5' primer	3' primer
D51E	GCCACCCCACAAGAGTTAAATACCATG	CATGGTATTTAACTCTTGTGGGGTGGC
D51Q	GCCACCCCACAACAATTAAATACCATG	CATGGTATTTAATTGTTGTGGGGTGGC
D51N	GCCACCCCACAAAATTTAAATACCATG	CATGGTATTTAAATTTTGTGGGGTGGC

The same mutations were also introduced into the HIV-1 molecular clone pNL4-3Δenv using the same mutagenic primers described above. QuickChange II XL site-directed mutagenesis kit (Stratagene) was used to create the point mutations in the *CA *sequence. All plasmid DNAs were then propagated in *E. coli *XL10-Gold and purified by Maxiprep Purification kit (Qiagen). The identity of each mutation was confirmed by sequencing and the resulting plasmids were digested with *BssH*II and *Apa*I. The 1295 bp *BssH*II/*Apa*I DNA fragments of the mutated *CA *sequences were then isolated, purified and cloned directionally into the pNL4-3 vector, digested with the same restriction enzymes. The resulting plasmids were propagated in DH5α competent *E. coli*, purified using Maxiprep purification kit and verified by sequencing.

### Capsid protein expression and purification

Competent *E. coli *Origami (DE3) cells were transformed with the three mutants or the wild-type pET11a-CA expression plasmid, expressed and purified essentially as described elsewhere [26]. Briefly, a single colony from a freshly streaked plate was initially grown in 50 ml LB-medium containing 100 μl Ampicillin (stock 100 mg/ml) and cultured at 37°C shaken at 220 r.p.m. Upon reaching optical cell densities at 600 nm (OD_600_) ~0.6–1.0, the cells culture was saved at 4°C overnight. The following day, 10 ml of culture was added to 1 litre of LB-medium containing ampicillin and incubated with shaking at 37°C until the OD_600 _was ~0.7–1.0. Protein expression was then induced by addition of isopropylthio-β-D-galactoside (IPTG) to a final concentration of 1 mM. After a 4 hrs incubation period at 37°C, the cells were harvested by centrifugation at 4000 r.p.m. for 10 min (Megafuge 2.0 R, rotor #8155, Kendro). The cell pellet was resuspended in 6 M Guanidine-HCl (pH 6.5) and stirred for 3 hrs at room temperature before being centrifuged at 10000 r.p.m. for 10 min at 4°C (Beckman Avanti J30-I, rotor 25.50, Beckman Coulter). Fifty ml of nuclease-free water was slowly added to the supernatant giving a final concentration of 1 M Guanidine-HCl to the protein solution. The protein solutions were put in four 15 cm long dialysis tubings (Spectrpor, MWCO 6–8000, 1.7 ml/cm) and dialyzed against 50 mM Tris pH 8.0 overnight at room temperature. Next, the contents of the dialysis tubings were pooled and centrifuged at 10000 r.p.m. for 10 min at 4°C (Beckman Avanti J30-I, rotor 25.50, Beckman Coulter) to remove precipitated proteins. The CAp24 proteins were then precipitated by addition of saturated (NH_4_)_2_SO_4 _to a final concentration of 30% and incubated on ice for 1 h. The CAp24 proteins were then collected by centrifugation at 20000 r.p.m. for 20 min at 4°C (Beckman Avanti J30-I, rotor 25.50, Beckman Coulter). Finally, the protein precipitate was dissolved in a buffer containing 50 mM Tris-HCl pH 8, 30 mM NaCl and 1 mM EDTA, and purified on ÄKTA FPLC chromatography system (Amersham Biosecience). The protein samples were initially loaded onto an anion-exchange column, HiTrap DEAE 1 ml FF, with a mobile phase of 50 mM Tris pH8.0, 30 mM NaCl, and 1 mM EDTA and flow rate of 1 ml/min. The absorbance was measured at 280 nm. The peak fractions containing the CAp24 proteins were pooled and precipitated with 50% saturated (NH_4_)_2_SO_4 _on ice for 1 h. The solution was then centrifuged at 20000 r.p.m. for 20 min at 4°C (Beckman Avanti J30-I, rotor 25.50, Beckman Coulter) and the precipitate was resupsended in 50 mM Tris pH8.0, 30 mM NaCl, and 1 mM EDTA. The purity and integrity of each CAp24 protein was finally analyzed by SDS-PAGE. In order to increase the purity of the CAp24 protein, the samples were loaded onto a gel filtration column, HiLoad 16/60 Superdex 75 prep grade, and run with the same mobile phase and as above but with a flow rate of 1.5 ml/min. The peak fractions containing the CAp24 proteins were pooled and concentrated by Amicon Ultra Centrifugal filters (Millipore; MWCO 5 k) and saved in aliquots at -80°C. A small aliquot (10 μl) was run on SDS-PAGE gel and the protein concentration was determined with a Bio-Rad *DC *Protein Assay Kit.

### Transfection procedure

Transfection was performed in a 6-well culture plate using the non-liposomal FuGENE 6 transfection reagent (Roche). Approximately 1 × 10^5 ^adherent cells (HeLa-tat, 293T, and COS7) were seeded one day before and transfected with 2 μg of each plasmid DNA mixed with 6 μl FuGENE 6 transfection reagent. Forty-eight to seventy-two hrs post-transfection, cells were washed in cold PBS and harvested in 1× RIPA buffer [50 mM Tris (pH 7.4), 150 mM NaCl, 1% Triton X-100, 1% Na-deoxycholate, and 0.1% SDS] supplemented with a complete protease inhibitor cocktail obtained from Roche.

### Virus stock preparation

Wild type and mutant virus stocks were prepared essentially as described before [27]. Briefly, HeLa-tat, COS7, and 293T cells were transfected as described above and three days post-transfection, culture supernatants were clarified from cell debris by centrifugation at 1200 r.p.m. for 7 min, and filtered through 0.45 μm filters. Cleared culture supernatants were then treated or not with DNase I (Roche Applied Science) at 20 μg/ml final concentrations at 37°C for 1 h and saved at -80°C until needed. The CAp24 antigen contents of each culture supernatant was determined by an in-house HIV-1 CAp24 antigen ELISA as previously described [27,28].

### Virus precipitation

HeLa-tat, COS7, and 293T cells were transfected with the wild type and mutant proviral DNAs as described above. Approximately seventy-two hrs post-transfection, virion-associated viral proteins were prepared from cell culture supernatants by removal of cellular debris by centrifugation at 1 200 r.p.m. for 7 min and filtering through a 0.45-μm-pore-size membrane. The virus particles in the culture supernatants were then concentrated by centrifugation using Viraffinity (CPC Inc.) essentially as described before [29]. Briefly, clarified culture supernatants were mixed with Viraffinity (3:1) and the mixture was incubated at room temperature for 5 min. They were then centrifuged at 1 000 × g for 10 min and viral pellets washed twice in a buffer containing 60 mM HEPES, 150 mM NaCl, pH 6.5. Finally, the viral pellets were dissolved in 1× RIPA buffer, mixed with 2× SDS sample buffer and boiled for 5 min before being subjected to sodium dodecyl sulphate-polyacrylamide gel electrophoresis (SDS-PAGE).

### Western blot

Denatured whole cell extracts or viral lysates were separated on 10–20% SDS-PAGE gels (Invitrogen), transferred onto a nitrocellulose membrane (Amersham Bioscience) overnight at 4°C and detected either with monoclonal anti-CAp24 antibody (kindly provided by Dr Hinkula J), polyclonal anti-CAp24, anti-cyclophilin A, anti-calnexin (Santa Cruz) antibodies or a cocktail of three different HIV-1 positive human sera. As a secondary antibody, appropriate horseradish peroxidase-conjugated anti-mouse (DAKO; 1:4000), anti-rabbit (Sigma; 1:40000), or anti-human (Pierce; 1:20 000) IgG antibody was used.

### Viral infectivity assay

The mutant and wild type HIV-1 virus stocks were prepared as described above and 100 ng CAp24 antigen equivalents were used to infect MT4 cells. Briefly, 1 × 10^5 ^cells were infected with normalized amounts of virus for 3 hrs at 37°C. The cells were then pelleted, residual virus was removed, and the cell cultures were incubated in fresh complete medium supplemented with FBS and antibiotics at 37°C in 5% CO_2_. Three days post-infection, the CAp24 antigen contents in the culture supernatants were then processed for CAp24-ELISA.

### Single cell cycle infectivity assay

TZM-bl cells (6 × 10^4 ^cells per 12-well plate) [10] were seeded one day before infection. Following day, medium was removed and cells were infected with mutant and wild type NL4-3 virus. The cells were infected with a virus stock corresponding to 50 ng CAp24 antigen per well with 20 μg/ml DEAE-dextran in a total volume of 300 μl. After adsorption period of 3 hrs, input virus was removed and cells were fed with a complete DMEM containing 10 μM indinavir and cultured for 24 hrs. Finally, culture supernatants were removed and cells were lysed with 200 μl Glo lysis buffer (Promega). One-hundred μl of the cell lysates were then assayed for luciferase activity using the luciferase assay kit obtained from Promega as recommended by the manufacturer. Measurement of the luminescence was done using the Luminoskan Ascent luminometer (ThermoLabsystem).

### In vitro HIV-1 CA assembly (Turbidity assay)

Turbidity assay is a valuable technique used to study a salt-induced self-assembly process of CAp24 by monitoring polymerization of CAp24 spectrophotometrically, as the rate of CAp24 tube formation increases sample turbidity [9,30,31]. The assay was performed at room temperature using a BioSpec-1601E spectrometer (Shimadzu) and the absorbance was set to 350 nm wavelength. One-hundred μM of highly purified HIV-1 CAp24 protein of each mutant and the wild type control was mixed with 50 mM NaH_2_PO_4 _(pH 8.0). Tubular CAp24 assembly was then induced by addition of 2.0 M NaCl solution, and the assembly rates was monitored by a spectrophotometer as the rate of tube formation increases the sample turbidity. Absorbance measurements were made every 10 s for up to 60 min. The assembly rate was then set by plotting the absorbance versus time.

For TEM analysis, 100 μM of each mutant and the wild type CAp24 protein was mixed with 50 mM NaH_2_PO_4 _(pH 8.0) and 1.0 M NaCl solution. The mixture was then immediately transferred to a 37°C and incubated for 1 h. Finally, the samples were fixed with freshly made 2.5% formaldehyde and processed for TEM analysis.

### Immunofluorescence assay

HeLa-tat III cells (1.5 × 10^3 ^cells per well in 4-well chambered slides from Nunc) were cultured one day before and transfected with 2 μg of mutant and wild type proviral DNA constructs. Forty-eight hrs post-transfection, cells were fixed in aceton/methanol (1:1) for 5 min and washed with PBS. Slides were then incubated with primary anti-CAp24 monoclonal antibody and 4',6-diamidino-2-phenylindole dihydrochloride (DAPI) at 37°C for 1 h. DAPI was used to labell the cellular DNAs. Cells were washed three times in PBS and further incubated with secondary antibody for 1 h. FITC-conjugated rabbit anti-mouse IgG antibody (DAKO) was used as secondary antibody. After the final wash, slides were mounted and flourescent images were aquired by using a Nikon Eclipse E600 phase-contrast fluorescent microsope.

### Transmission electron microscopy analysis

Cells were prepared for electron microscopy essentially as described elsewhere [32]. Briefly, transfected HeLa-tat cells and virus infected Jurkat-tat cells (data not shown) were fixed by freshly made 2.5% glutaraldehyde in phosphate buffer and post-fixed in 1% OsO_4_. The cells were embedded in epon and post-stained with 1% uranyl acetate. Epon sections were cut at approximately 60 nm thick to accommodate the volume of the core structure parallel to the section plane. Duplicate sample preparations were done, which were then analyzed by electron microscope.

Additionally, in vitro assembled CAp24 proteins were negatively stained with 2% ammonium molybdate at pH 8.0 to study the CAp24 tubular formation.

## Competing interests

The author(s) declare that they have no competing interests.

## Authors' contributions

SA and MY contributed equally to the experimental work. SA wrote the manuscript with AV. AV is the principal investigator. SH performed all electron microscopy analysis. All authors read and approved the manuscript.
